# Dendritic cell-associated MAVS is required to control West Nile virus replication and ensuing humoral immune responses

**DOI:** 10.1371/journal.pone.0218928

**Published:** 2019-06-26

**Authors:** Kelsey Roe, Daniela Giordano, Lucy B. Young, Kevin E. Draves, Ursula Holder, Mehul S. Suthar, Michael Gale, Edward A. Clark

**Affiliations:** 1 Department of Immunology, University of Washington, Seattle, Washington, United States of America; 2 Emory Vaccine Center, Yerkes National Primate Research Center, Atlanta, Georgia, United States of America; 3 Department of Pediatrics, Division of Infectious Disease, Emory University School of Medicine, Atlanta, Georgia, United States of America; 4 Center for Innate Immunity and Immune Disease, University of Washington, Seattle, Washington, United States of America; University of Amsterdam, NETHERLANDS

## Abstract

Mitochondrial antiviral signaling protein (MAVS) is a critical innate immune signaling protein that directs the actions of the RIG-I-like receptor (RLR) signaling pathway of RNA virus recognition and initiation of anti-viral immunity against West Nile virus (WNV). In the absence of MAVS, mice die more rapidly after infection with the pathogenic WNV-Texas (TX) strain, but also produce elevated WNV-specific IgG concomitant with increased viral burden. Here we investigated whether there was a B cell intrinsic role for MAVS during the development of protective humoral immunity following WNV infection. MAVS^-/-^ mice survived infection from the non-pathogenic WNV-Madagascar (MAD) strain, with limited signs of disease. Compared to wildtype (WT) controls, WNV-MAD-infected MAVS^-/-^ mice had elevated serum neutralizing antibodies, splenic germinal center B cells, plasma cells and effector T cells. We found that when rechallenged with the normally lethal WNV-TX, MAVS^-/-^ mice previously infected with WNV-MAD were protected from disease. Thus, protective humoral and cellular immune responses can be generated in absence of MAVS. Mice with a conditional deletion of MAVS only in CD11c^+^ dendritic cells phenocopied MAVS whole body knockout mice in their humoral responses to WNV-MAD, displaying elevated virus titers and neutralizing antibodies. Conversely, a B cell-specific deletion of MAVS had no effect on immune responses to WNV-MAD compared to WT controls. Thus, MAVS in dendritic cells is required to control WNV replication and thereby regulate downstream humoral immune responses.

## Introduction

The mosquito-borne neurotropic flavivirus West Nile virus (WNV) is the most important cause of epidemic viral encephalitis in North America. Although neutralizing antibodies (nAbs) are essential for protection after infection with WNV or related flaviviruses such as Dengue virus and Zika virus, relatively little is known about what early events after flavivirus infection are required in innate immune cells to program protective Ab responses. Flavivirus infection is recognized by the RIG-I-like receptor (RLR) pathogen recognition receptor proteins, RIG-I and MDA5 [1,2]. The RLRs bind viral RNA and undergo activation events to then bind to the signaling adaptor protein MAVS. RLR binding to MAVS induces signaling to activate downstream transcription factors that direct the expression of immune modulatory genes, antiviral genes, and the production of types I and III interferon (IFN) [3–5]. IFN then induces the expression of interferon stimulated genes (ISGs). ISG products have antiviral and immune-modulatory actions that serve to regulate the onset and actions of the adaptive immune responses. Innate immunity, and both humoral and T cell immunity are essential for the control of WNV infection and neuropathogenesis [1,4,6]. Type I IFN receptor (IFNAR) and type III IFN receptor are expressed on a variety of cell types and are signaled in turn to activate ISG expression across a variety of tissues, leading to strong anti-viral responses and programming of adaptive immunity [3,6,7].

C57BL/6 (WT) mice infected with WNV via footpad injection, to model a mosquito bite, develop clinical disease similar to that seen in WNV-infected humans that can range from a subclinical infection to encephalitis and death. The type I and type III IFN pathways play key roles for protective immunity in WT mice [3,5]; mice deficient in IFNAR or IFN response factors IRF3, IRF5 or IRF7 develop severe disease and succumb to infection [1,8]. MAVS plays a key role in type I IFN-dependent protective immunity against WNV. MAVS-deficient (MAVS^-/-^) mice infected with a pathogenic strain of WNV, WNV-Texas (TX), uniformly died from infection within 8–9 days post infection (p.i.) [9]. MAVS deficiency led to increased virus replication, early dissemination of WNV-TX into the central nervous system (CNS) and dysregulated inflammation including elevated levels of type I IFN and inflammatory cytokines [9,10]. A recent study suggested that MAVS expressed in hematopoietic cells is critical to control WNV infection and pathogenesis [10]. Just specifically what cell types require MAVS to resist WNV remain unclear.

Strikingly, WNV-TX-infected MAVS^-/-^ mice had elevated levels of virus-specific Ab responses; six days after infection IgG Ab levels were about 200 times higher in infected MAVS^-/-^ mice than wildtype (WT) mice [9]. Surprisingly, despite having much higher levels of virus-specific Abs, the nAb titers in MAVS^-/-^ mice were not that much higher than titers detected in infected WT mice. This result suggested that MAVS regulates not only the quantity of IgG Ab responses to WNV, but also the quality of the Ab response, such as Ab affinity. However, we were not able to examine the role of MAVS in B cell responses such as germinal center (GC) formation since MAVS^-/-^ mice died by 9 days after WNV-TX infection.

To explore the role of MAVS in anti-viral humoral immunity we made use of an WNV isolate, WNV Madagascar 78 (WNV-MAD), which is not pathogenic in immunocompetent mice [11]. Here we report that MAVS^-/-^ mice, unlike after infection with WNV-TX, can resist infection by an infectious clone (i.c.) of WNV-MAD [12] and after WNV-MAD infection develop immunity to otherwise lethal WNV-TX. After WNV-MAD infection Ab responses and viral titers were significantly higher in MAVS^-/-^ mice than in WT mice. Importantly, conditional knockout (cKO) mice with MAVS-deficiency restricted to CD11c^+^ dendritic cells (DCs) had a similar phenotype as MAVS^-/-^ mice, underscoring the critical role MAVS plays in DCs in programming resistance to WNV and probably other viruses.

## Materials and methods

### Mice

C57BL/6J (WT) mice were purchased from Jackson Labs (Bar Harbor, ME). MAVS^-/-^ (C57BL/6x129Sv/Ev) mice were kindly proved by Dr. S. Akira (Osaka University, Osaka) [13]. MAVS^*fl/fl*^ mice (on the B6/J background) were developed at the University of California at Davis Mouse Biology Program (see S1 Fig). CD11c^Cre+^ mice (on a B6/J background), developed by Caton et al. [14], were kindly provided by Dr. Mike Bevan (University of Washington, Seattle WA). The Mb1^Cre+^ mice (on the B6/J background) [15], with permission from Dr. Michel Reth, were kindly provided by Dr. Tony DeFranco (University of California San Francisco). Mice for experiments were 8–12 weeks old, were sex-matched, and were housed in a specific pathogen free environment.

### Ethics statement

This study was carried out in strict accordance with the recommendations in the Guide for the Care and Use of Laboratory Animals of the National Institutes of Health. All procedures were approved and conducted according to regulations of the Institutional Animal Care and Use Committee of the University of Washington, Seattle, WA (IACUC, Protocol #2242±08). Footpad injections were performed under anesthesia that was induced and maintained with ketamine hydrochloride and xylazine, and all efforts were made to minimize suffering.

### West Nile virus infections

Non-pathogenic lineage 2 WNV-MAD i.c., derived from the Madagascar AgMg789 strain, and the pathogenic lineage 1 WNV-TX i.c., derived from the Texas 2002-HC strain, were described previously and were propagated in Vero cells [11]. Infectious clones were produced from each virus, and stock titers were determined by a plaque assay using BHK-21 cells [12]. For *in vivo* infections, mice were inoculated under anesthesia with 100 PFU WNV-MAD i.c. subcutaneously into the footpad in a total of 20 μL. For challenge studies, mice were infected with 1000 PFU WNV-TX five weeks after WNV-MAD infection. Serum was isolated from blood, collected via the retro-orbital route every 7 days, and stored at -80°C until use.

### Mouse survival and monitoring

Following lethal WNV-TX infection, mice were monitored at least once daily, twice during peak disease, for body weight and clinical signs of disease and distress. Clinical scores were established as; 1: ruffled fur, lethargic, or hunched, no paresis; 2: very mild to mild paresis; 3: frank paresis involving at least 1 hind limb, or conjunctivitis or mild paresis in both hind limbs; 4: severe paresis, still retains feeling, possibly limbic; 5: paralysis; 6: moribund. Mice that had lost more than 20% of their original body weight or were determined to be a clinical score of 5 or 6 were euthanized immediately. A total of 62 mice received lethal WNV-TX and were monitored for the duration of the experiment of 21 days. Despite careful monitoring, 5 mice were found dead; 14 mice were euthanized during the study having met endpoint criteria.

### WNV RNA quantitation

Whole spleens were harvested from euthanized mice following WNV-MAD infection. Splenocytes were isolated by mechanical separation between frosted glass slides and red blood cells were lysed (BioLegend). RNA was extracted from lysed splenocytes using a Qiagen RNAeasy mini kit. WNV-specific cDNA was created with a high capacity cDNA kit (AppliedBiosystems) using a WNV reverse primer, and qRT-PCR was performed using TaqMan GeneExpression master mix (AppliedBiosciences) and primers (Forward: CCTGTGTGAGCTGACAAACTTAGT, Reverse: GCGTTTTAGCATATTGACAGCC), a FAM/TAMRA probe (6 FAM-CCTggTTTCTTAgACATCgAgATCTXCgTgCp) and protocol described by Linke et al. [16].

### ELISA and ELISPOT

Sera from naïve or WNV-MAD infected mice were inactivated by ultraviolet light 2x10^5^ μJ/cm^2^ for 30 min, followed by heat inactivation at 56°C for 30 min. WNV envelope protein (WNVE)-specific IgM or IgG was quantitated by ELISA assay as previously described [17]. Briefly, polystyrene plates were coated with recombinant WNVE protein, derived from lineage 1 WNV New York 2000 strain and generously provided by Dr. Michael Diamond (Washington University, St. Louis MO) [18]. Plates were blocked with 5% bovine serum albumin, followed by incubation with dilutions of sera. Plates were washed with phosphate buffered saline (PBS) plus 0.05% Tween-20 and developed using anti-mouse IgM or anti-mouse IgG horseradish peroxidase (HRP) secondary antibodies followed by AEC substrate. IgM or IgG levels were quantitated by comparison to a standard curve of known Ig concentrations. For ELISPOTS, dilutions of isolated splenocytes or bone marrow cells were incubated on WNVE-coated cellulose ester membrane filter plates (Millipore) overnight at 37°C and IgM or IgG spots were detected using αIgM- or αIgG-HRP and AEC substrate as previously described [17].

### FRNT_50_

Titers of WNV neutralizing Abs were quantitated by a focus forming reduction neutralization test (FRNT). Two-fold serial dilutions of sera, that were previously UV and heat inactivated, were prepared in DMEM media (GenClone) containing 5% fetal bovine serum (FBS, ThermoFisher) and L-glutamate, penicillin and streptomycin (Corning). WNV-TX was diluted in DMEM and mixed with the diluted serum, reducing the dilutions by half, and incubated at 37°C for one hr to allow for neutralization. 96-well plates of Vero cells at 80–90% confluency were then infected with the serum/virus mixtures, including several wells with virus only. One hour after infection at 37°C, a 1% methylcellulose overlay was added, consisting of one part 2% methylcellulose in PBS and one part 2XDMEM media. Cells were then incubated for 25 hrs, after which they were gently washed in PBS and fixed with 4% formaldehyde (Sigma) for 20 min. Cells were then washed and stored in PBS at 4°C until staining. Cells were permeabilized with 0.1% saponin (Sigma) in PBS, blocked with 5% non-fat milk in PBS and stained with an αflavivirus antibody (clone 4G2, Millipore) at 37°C. Cells were washed in PBS, stained with αIgG-HRP at 37°C and developed with KPL TrueBlue peroxidase substrate (SeraCare). Spot numbers were quantified using a CTL-ImmunoSpot S5 Core Analyzer ELISPOT reader with ImmunoSpot Academic version 5.0 software (Cellular Technology Ltd.). The FRNT_50_ titers were calculated as the lowest dilution of serum with less than 50% of the average number of spots in the virus only wells.

### Flow cytometry

Splenocytes were processed into single cell suspensions as above and stained for flow cytometry analysis as previously described [17]. Briefly, cells were incubated with a fixable viability dye (Molecular Probes) in the absence of FBS, to discriminate dead cells. Cells were then blocked using an anti-F_C_ receptor antibody (BioLegend) and stained for surface markers (see S1 Table) and then fixed in 2% paraformaldehyde. Cells were processed with an LSRII flow cytometer (BD) and data was analyzed using FlowJo (v.10, Tree Star). See S2 Fig for gating strategies.

### Statistical analysis

Survival data were analyzed by Mantel-Cox log-rank test. Timeline data were analyzed using a 2-way ANOVA with Sidak’s multiple comparison test. Analyses between two groups were performed by Mann-Whitney test. Data from cKO mice were compared to their own littermate controls using the Mann-Whitney test. Analyses between four groups were performed by Kruskal-Wallis test with Dunn’s multiple comparison test. All statistical analyses were performed using GraphPad Prism 7. Differences of *p*<0.05 were considered significant.

## Results

### MAVS^-/-^ mice survive after infection with the non-pathogenic WNV strain, WNV-MAD

**Fig 1:**
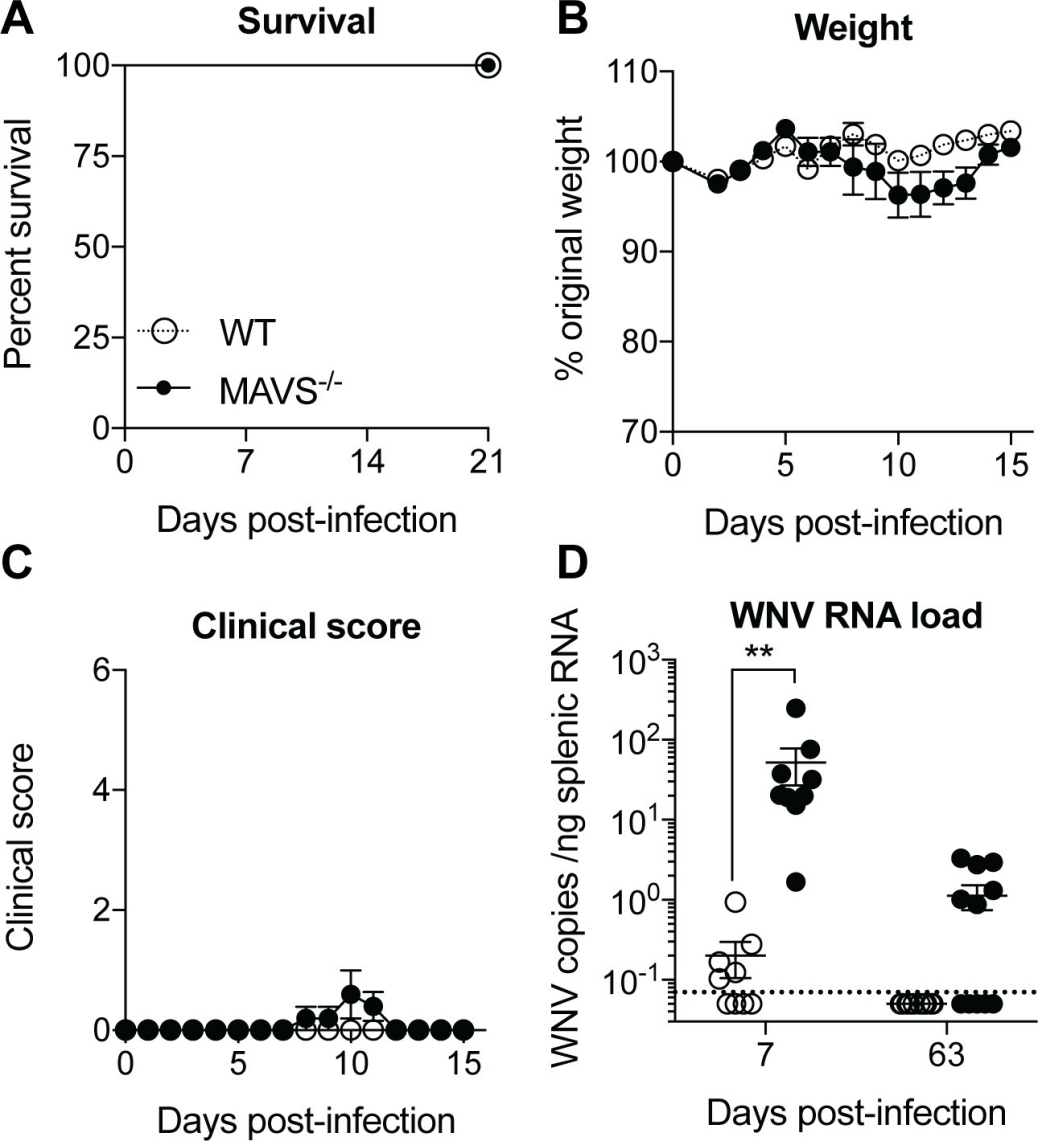
Mice deficient in MAVS display elevated levels of virus following infection with WNV-MAD. WT and MAVS^-/-^ mice were infected with 100 PFU WNV-MAD via the footpad. Mice were evaluated for (A) survival, (B) weight and (C) clinical score. Data are representative of three different experiments; n = 4 (WT), n = 5 (MAVS^-/-^). (D) WNV copy numbers were evaluated by qRT-PCR from RNA extracted from RBC-lysed splenocytes. The dotted line represents the limit of detection. Data are combined from two (D7) or three (D63) experiments; n = 9–11. ** *p*<0.01 by 2-way ANOVA with Sidak’s multiple comparison test.

MAVS^-/-^ mice all succumb to pathogenic lineage 1 WNV-TX infection within 8–9 days due to their inability to control virus replication and spread into the central nervous system [9]. In order to investigate the development of long-term humoral responses to WNV in the absence of MAVS and to address if MAVS plays an intrinsic role in B cells after WNV infection, we first tested whether or not MAVS^-/-^ mice could survive infection of a less pathogenic form of WNV, WNV-MAD [11,12]. After footpad inoculation with 100 PFU of WNV-MAD, MAVS-deficient mice survived infection and showed only occasional mild disease symptoms (Fig 1A–1C). Nonetheless, WNV-MAD replicates well in the absence of MAVS, with almost 100-fold more virus detected in MAVS^-/-^ mice in the spleen 7 days after infection compared to WT mice (Fig 1D). In addition, WNV RNA was still detected in the spleens of MAVS^-/-^ but not WT mice 63 days after infection (Fig 1D).

### MAVS signaling limits the generation of B cell and T cell responses following WNV-MAD infection

**Fig 2:**
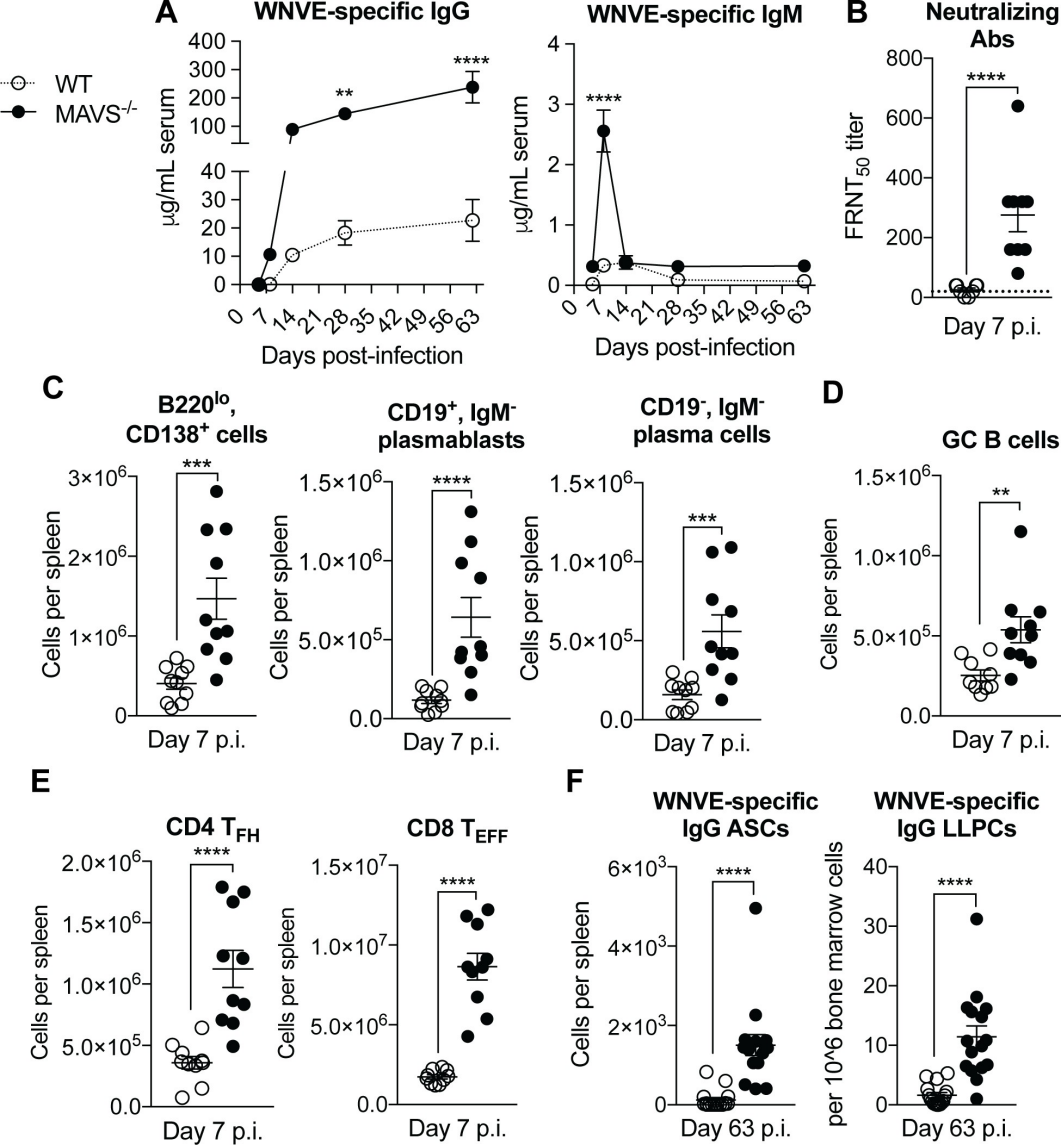
Absence of MAVS results in elevated WNVE-specific Ab and plasma cell responses. WT and MAVS^-/-^ mice were infected with 100 PFU WNV-MAD. (A) WNVE-specific IgG from sera were analyzed by ELISA. (B) Neutralizing Abs were analyzed by FRNT_50_ in vero cells. The dotted line indicates the limit of detection. (C) Flow cytometric analysis of CD3^-^, B220^lo^, CD138^+^ cells, IgM^-^, CD19^+^ plasmablasts and IgM^-^, CD19^-^ plasma cells from total splenocytes. (D) B220^+^, CD38^-^, GL7^+^ GC B cells were evaluated from total splenocytes by flow cytometry. (E) Flow cytometry analysis of CD4^+^ T follicular helper cells (CD4 T_FH_), defined as CD3^+^, CD4^+^, CD44^+^, CD62L^-^, CXCR5^+^, PD-1^+^ and CD8^+^ T effector cells (CD8 T_EFF_), defined as CD3^+^, CD8^+^, CD44^+^, CD62L^-^. p.i. = post infection. (F) WNVE-specific IgG Ab secreting cells (ASCs) from total splenocytes or long-lived plasma cells (LLPCs) from bone marrow by ELISPOT. Data is representative of three experiments (A) or combined from two (B,C,D,E) or four (F) experiments. *p<0.05, **p<0.01, ***p<0.001, ****P<0.0001 by 2-way ANOVA with Sidak’s multiple comparison test (A) or by Mann-Whitney test (B-F).

We next investigated the role of MAVS in the development of humoral responses during WNV infection. Following infection with WNV-MAD, MAVS^-/-^ mice developed significantly higher serum WNVE-specific IgM and IgG than WT mice (Fig 2A). Interestingly, we observed a significant increase in the levels of nAbs in MAVS^-/-^ mice at day 7 post-infection (p.i.), at a time point were nAbs were mostly undetected in WNV-MAD infected WT mice (Fig 2B). As these data would predict, we found significantly elevated B220^lo^ CD138^+^ plasma cells in MAVS^-/-^ mice compared to WT mice following WNV-MAD infection as early as day 7 and up to day 10 p.i. (Fig 2C and S3A Fig). In particular, after infection MAVS^-/-^ mice had significant increases specifically in the IgM^-^ cells, both CD19^+^ plasmablasts and CD19^-^ plasma cells (Fig 2C); these cells are most likely IgG-secreting. Correspondingly, we observed significant increases in WNVE-specific IgM and IgG Ab secreting cells (ASCs) in the spleen at day 7 (IgM and IgG) and day 10 (IgG only) p.i. (S3B Fig). Intriguingly, MAVS^-/-^ mice also had elevated levels of GC B cells compared to WT mice at 7- and 10-days post WNV-MAD infection (Fig 2D and S3C Fig). These data were consistent with the observed increase in nAbs in MAVS^-/-^ mice at day 7 p.i..

As we observed robust GC responses after infection in the absence of MAVS, we next asked if T follicular helper cells (T_FH_), necessary to help affinity maturation of B cells in the germinal center, were also elevated. This was indeed the case (Fig 2E); we detected an over 2-fold increase in T_FH_ cells in MAVS^-/-^ mice compared to WT mice 7 days after WNV-MAD infection. Furthermore, we observed a significant increase in the numbers of CD8^+^ T effector cells (T_EFF_) in the spleens of MAVS^-/-^ mice versus WT mice (Fig 2E). Finally, although the GCs in both WT and MAVS^-/-^ mice contracted significantly over the course of a nine-week infection (S3C Fig), WNVE-specific IgG ASCs in the spleen and long-lived plasma cells (LLPCs) in the bone marrow (BM) were still significantly elevated in MAVS^-/-^ mice versus WT at this time point (Fig 2F), mirroring serum IgG levels (Fig 2A).

### Non-pathogenic WNV infection induces protective immune responses in mice deficient for MAVS

**Fig 3:**
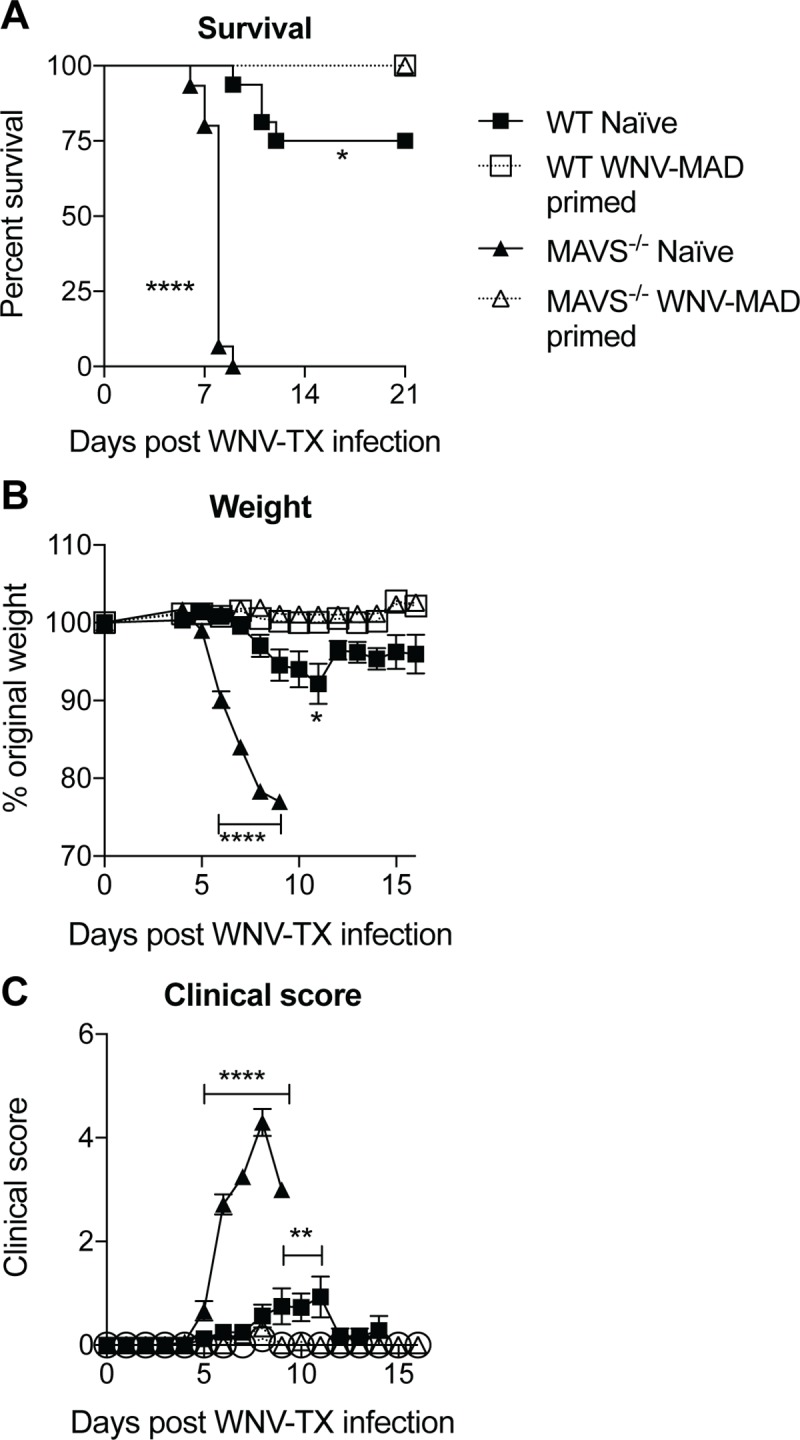
A single inoculation of WNV-MAD is sufficient to protect MAVS^-/-^ mice from lethal WNV-TX disease. WT and MAVS^-/-^ mice were infected via footpads with 100 PFU WNV-MAD. One month later naïve and WNV-MAD-primed mice were infected with 1000 PFU WNV-TX. (A) Survival, (B) weight and (C) clinical scores were evaluated. Data are from combined from two independent experiments; n = 15 each group. *** *p*<0.001, **** *p*<0.0001 by (A) Mantel-Cox log-rank test or (B,C) 2-way ANOVA with Sidak’s multiple comparison test between naïve and WNV-MAD primed mice within each genotype.

One open question that could not be tested with lethal WNV strains, was whether the enhanced adaptive immune responses induced in MAVS^-/-^ mice after WNV infection were protective. Therefore, we asked whether the combined cellular and humoral responses observed in the MAVS^-/-^ mice following the WNV-MAD infection were sufficient to protect them from a lethal WNV-TX infection. We challenged naïve WT and MAVS^-/-^ mice and mice that had been infected with WNV-MAD 5 weeks previously with a lethal dose of WNV-TX (Fig 3A). While approximately 25% of WT and 100% of MAVS^-/-^ naïve mice succumbed to WNV-TX infection by day 9, all of the WNV-MAD infected mice survived a lethal challenge. Furthermore, both WT and MAVS^-/-^ mice were protected from weight loss and disease if they had been previously infected with WNV-MAD (Fig 3B and 3C).

### MAVS in dendritic cells but not in B cells influences humoral immune responses to WNV infection

To determine whether there was any intrinsic role for MAVS in B cells, or conversely test that virus recognition and control by DCs was critical during the development of humoral immunity to WNV, we generated MAVS conditional knockout (cKO) mice. MAVS^fl/fl^ mice were generated at the Mouse Biology Program at the University of California at Davis (S1A Fig). We crossed these mice with CD11c^Cre+^ or Mb1^Cre+^ mice, generating mice deficient in MAVS in dendritic cells (MAVS^fl/fl^CD11c^Cre+^) or B cells (MAVS^fl/fl^Mb1^Cre+^). The expression of MAVS mRNA in purified CD11c^+^ DCs and B cells confirmed that exon 3 of MAVS was knocked out appropriately in the respective MAVS cKO mice (S1B Fig).

**Fig 4:**
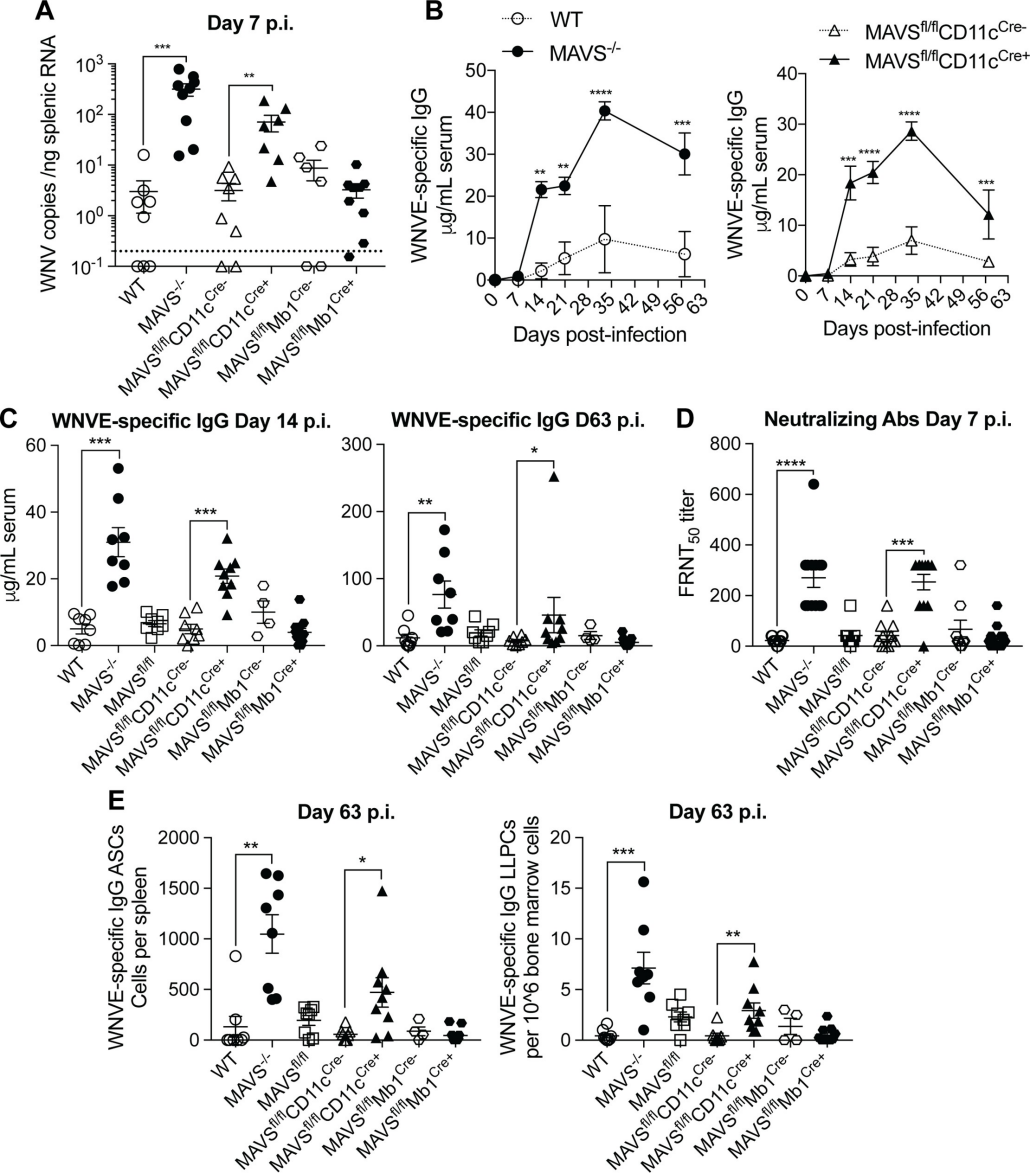
Mice with a deficiency of MAVS in CD11c^+^ cells phenocopy the elevated humoral responses of MAVS^-/-^ mice following WNV-MAD infection. WT, MAVS KO, MAVS^*fl/fl*^, MAVS^*fl/fl*^CD11c^Cre-^, MAVS^*fl/fl*^CD11c^Cre+^, MAVS^*fl/fl*^Mb1^Cre-^ and MAVS^*fl/fl*^Mb1^Cre+^ mice were infected with 100 PFU WNV-MAD. (A) WNV copy numbers were evaluated by qRT-PCR from RNA extracted from RBC-lysed splenocytes. The dotted line represents the limit of detection. (B,C) WNVE-specific IgG was quantitated by ELISA. (D) Neutralizing Abs were analyzed by FRNT_50_ in vero cells. (E) WNVE-specific IgG ASCs from spleen or LLPCs from bone marrow were analyzed by ELISPOT. p.i. = post infection. Data are representative of two experiments (B) or combined from two (A,C,E) or three experiments (D). *p<0.05, **p<0.01, ***p<0.001 by 2-way ANOVA with Sidak’s multiple comparison test (B) or by Mann-Whitney test between WT and (c)KO mice (A,C-E).

We first infected these mice with WNV-MAD and assessed their spleens 7 days later for WNV virus loads (Fig 4A). The MAVS^fl/fl^CD11c^Cre+^ mice had significantly elevated WNV RNA loads as compared to their littermate controls (MAVS^fl/fl^CD11c^Cre-^) or MAVS^fl/fl^ mice, much like the MAVS^-/-^ mice versus WT mice (Fig 1). Conversely, the MAVS^fl/fl^Mb1^Cre+^ mice had similar virus titers as their littermate controls (MAVS^fl/fl^Mb1^Cre-^), and similar to WT mice. Furthermore, MAVS^fl/fl^CD11c^Cre+^ mice also had elevated WNVE-specific IgG titers compared to their littermate controls, phenocopying the differential kinetics between WT and whole-body MAVS^-/-^ mice (Fig 4B); we did not observe any differences in WNVE-specific IgG between MAVS^fl/fl^Mb1^Cre+^ and their littermate controls; they both expressed similar antibody levels as the parental MAVS^fl/fl^ and WT mice (Fig 4C). These data suggest that the control of WNV replication by DCs is critical in regulating the humoral response to the virus. In support of this conclusion, mice deficient in MAVS in DCs induced high titers of neutralizing Abs, while mice missing MAVS in B cells did not (Fig 4D). Furthermore, WNVE-specific IgG ASCs in the spleen and LLPCs in the BM 9 weeks after infection were also elevated in MAVS^fl/fl^CD11c^Cre+^ mice but not in MAVS^fl/fl^ or MAVS^fl/fl^Mb1^Cre+^ mice (Fig 4E).

**Fig 5:**
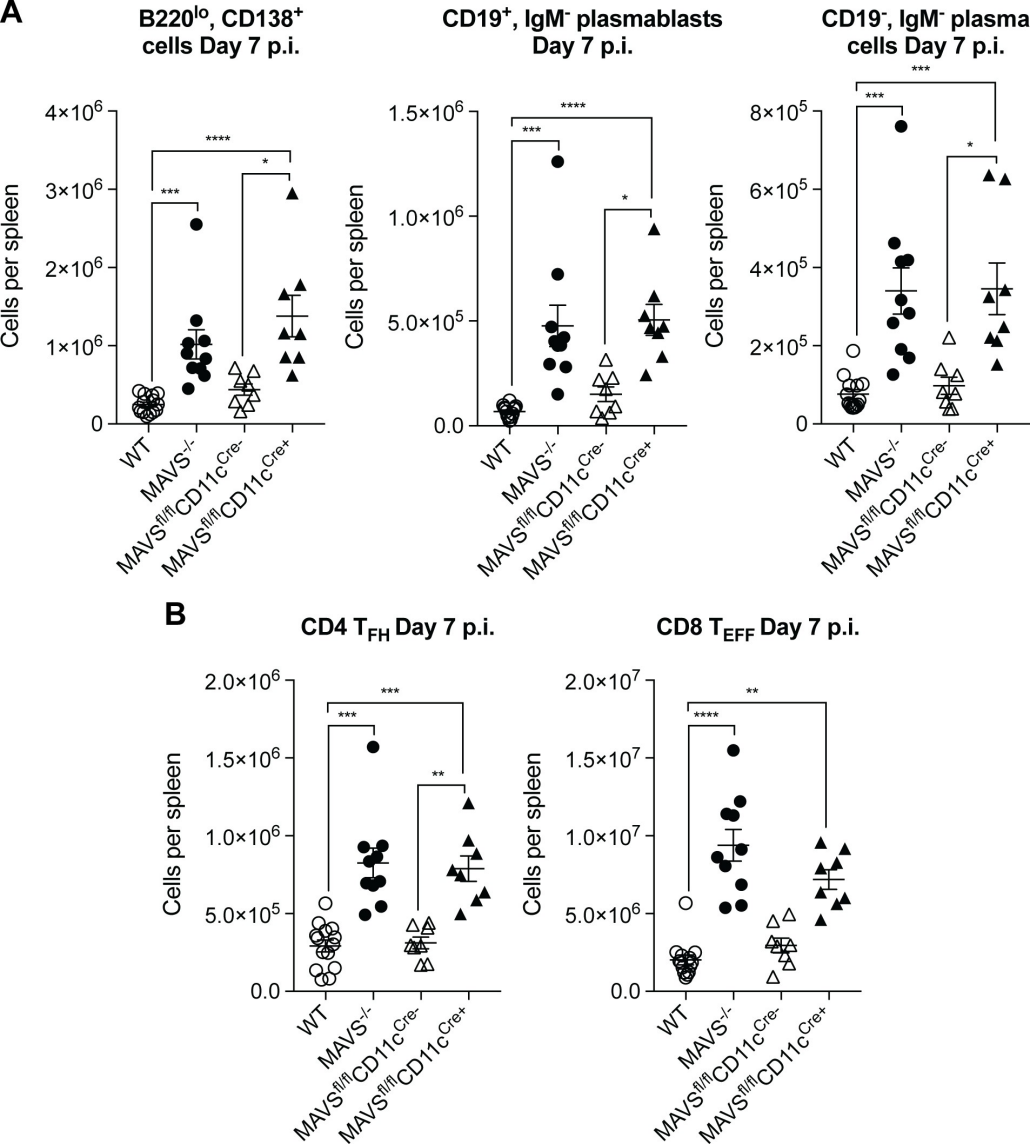
WNV infection induces increased numbers of plasma cells, T_FH_ cells and CD8^+^ T effector cells when MAVS is conditionally deleted from CD11c^+^ cells. WT, MAVS KO, MAVS^*fl/fl*^, MAVS^*fl/fl*^CD11c^Cre-^, MAVS^*fl/fl*^CD11c^Cre+^, MAVS^*fl/fl*^Mb1^Cre-^ and MAVS^*fl/fl*^Mb1^Cre+^ mice were infected with 100PFU WNV-MAD. (A) CD3^-^, B220^lo^, CD138^+^ cells, IgM^-^, CD19^+^ plasmablasts and IgM^-^, CD19^-^ plasma cells from total splenocytes were evaluated by flow cytometry. (B) CD4^+^ T follicular helper cells (CD4 T_FH_), and CD8^+^ T effector cells (CD8 T_EFF_), defined as in Fig 2, were evaluated from the spleen by flow cytometry. Data are combined from two individual experiments. *p<0.05, **p<0.01, ***p<0.001 by Kruskal-Wallis test with Dunn’s multiple comparison test.

Finally, we assessed splenic B lineage cells and T cells; we observed similar increases in IgM^-^ plasmablasts and plasma cells in MAVS^fl/fl^CD11c^Cre+^ mice and MAVS^-/-^ mice (Fig 5A). These data suggest that elevated virus replication in the absence of MAVS induces in the activation of more plasma cells, resulting in elevated serum WNV-specific IgG. Thus, we found no evidence for an effect of MAVS on the production of IgG on a per cell basis. Furthermore, mice lacking MAVS just in DCs also had elevated CD4^+^ T_FH_ cells and CD8^+^ T_EFF_ cells, as in MAVS^-/-^ mice (Fig 5B).

## Discussion

In this study we evaluated the role that the innate adaptor molecule MAVS plays in the development of humoral immune responses during WNV infection. In order to study the long-term antibody responses during WNV infection in the absence of MAVS, we first determined that MAVS-deficient mice can survive a non-pathogenic WNV-MAD infection (Fig 1). In contrast, IFNAR-deficient mice die after footpad inoculation with 100 PFU of WNV-MAD [11]; furthermore, WNV-MAD can be lethal when inoculated intracranially into WT mice [19,20]. The fact that MAVS^-/-^ mice control WNV-MAD infection and IFNAR^-/-^ mice do not [11], is most likely due to the fact that MAVS^-/-^ mice still make type I IFN after WNV infection, possibly downstream of TLR activation, and thus, presumably utilize the IFNAR pathway for protection [9].

Previously, we reported that 6 days after WNV-TX infection MAVS^-/-^ mice had about 200-fold higher levels of serum WNV-specific IgG compared to WT mice [9]. However, the same mice only had about 4-fold higher nAb levels compared to WT mice. This suggested that MAVS^-/-^ mice had a reduced capacity for Ab affinity maturation and possibly GC formation. We hypothesized that MAVS may play a role in the development of a robust GC response, such that WNV-specific Abs in MAVS-deficient mice would develop in extrafollicular (EF) regions and not undergo affinity maturation and selection in GCs. Consistent with this hypothesis, the addition of a RIG-I agonist to an inactivated influenza vaccine improves GC responses and protection from influenza infection [21]. While we did observe higher WNVE-specific IgG titers in MAVS^-/-^ compared to WT mice following WNV-MAD infection (Fig 2A), consistent with observations previously reported with WNV-TX [9], we also observed elevated nAbs, GC B cells and T_FH_ cells in the absence of MAVS (Fig 2B, 2D and 2E). Thus, the early expansion of GCs did not support our hypothesis of an initial dominant EF Ab response in the MAVS-deficient mice after WNV infection. However, our results do not exclude the possibility that both EF and GC responses might take place. Indeed, we previously found that WNV-TX infected B cell activating factor receptor (BAFFR)^-/-^ mice, which lack mature B cells, fail to induce GCs but nonetheless develop a protective nAb response [17]. In line with these results, the type I IFN signaling pathway downstream of MAVS has also been shown to inhibit differentiation of T_FH_ cells in STAT3^-/-^ mice following viral infection [22].

Our data are consistent with previous observations that WNV-specific IgG responses are predominantly dependent on CD4^+^ T cells [23]. Thus, in the absence of MAVS the elevated levels of IgG nAbs against WNV correlate with increases in both GC B cells and T_FH_ cells. Significantly higher levels of nAbs were also found in the absence of IFNα/β receptors during primary or secondary influenza virus infection [24]. Furthermore, our observation of elevated splenic CD8^+^ T_EFF_ cells in the absence of MAVS during WNV-MAD infection are consistent with the expansion of CD8^+^ T cells in the periphery and CNS previously observed in MAVS^-/-^ mice infected with a lethal WNV strain [9]. Critically, CD8^+^ T cell responses are essential to control viral spread in the spleen and CNS [18]. Taken together, the enhanced cellular and humoral responses observed in the absence of MAVS signaling the first week after WNV-MAD infection are likely due to the inability of MAVS^-/-^ mice to control viral replication, as shown by the substantial increase in virus found in spleen 7 days after infection (Fig 1D). Furthermore, it is possible that the persistence of virus RNA in the spleens of MAVS^-/-^ mice (Fig 1D) potentially serve as an innate immune pathogen associated molecular pattern (PAMP), which may perpetuate small GCs that we were unable to detect, and result in the lasting elevation of IgG ASCs in the spleen of MAVS^-/-^ mice 63 days after WNV-MAD infection (Fig 2F). Conversely, the lasting plasma cells in the absence of MAVS may reflect the larger number of plasma cells induced during the peak GC response between day 7 to 10 p.i.

Our data demonstrating that MAVS^-/-^ mice survive WNV-TX infection if they have been previously infected with WNV-MAD (Fig 3), suggest that even under conditions where type I IFN-driven innate immune responses are disrupted in the absence of MAVS signaling, enhanced adaptive immune responses can protect mice from a lethal WNV disease. Several studies have shown that B cell responses and humoral immunity are essential for protection from a lethal WNV infection [25–27]. In particular, the production of an early neutralizing IgM response controls viremia and triggers a protective IgG response that limits virus spread and lethal encephalitis [25,26]. Passive transfer of immune sera can protect B cell- and Ab- deficient (μMT) mice that otherwise succumb to infection [25]. Thus, enhanced WNV-specific Ab and nAb responses might be a major factor protecting against lethal WNV in WNV-MAD infected MAVS^-/-^ mice. However, given that CD8^+^ T cells are important for clearing infection from tissues and preventing viral persistence [18], they might also play an important role in protection of WNV MAD-infected MAVS^-/-^ mice after WNV-TX challenge. Our results are consistent with a recent study showing that MAVS^-/-^ mice that survived a primary infection of WNV with a nonstructural protein mutation (NS4B-P38G) were all protected from a lethal WNV challenge [28].

Type I IFN, downstream of RLR and MAVS signaling, potently enhances humoral immunity and promotes class-switch recombination by stimulating DCs *in vivo* [29]. Multiple studies have demonstrated a direct role for the RLR pathway and MAVS in influencing humoral immune responses [3,21,30,31]. For example, Influenza specific ASCs and the generation of high affinity Abs can be enhanced by activating the RIG-I/MAVS pathway [21]. Administration of a MAVS peptide fused to a membrane permeable domain from the HIV Tat protein, induced production of Type I IFN and contributed to protection from lethal influenza infection [30]. Furthermore, RIG-I sensing of RNA and MAVS signaling are required for the induction of optimal GCs in response to sheep red blood cell inoculation [31]. Thus, we were surprised to find heightened GC responses in mice deficient in MAVS. However, the enhanced GC formation and virus-specific humoral responses could be explained by the elevated levels of WNV, and therefore Ag, in the mice deficient for MAVS. Uncontrolled virus replication in the absence of MAVS results in the elevation of inflammatory cytokines [9], and thus may result in heightened activation of adaptive immunity.

Overall, our data show that eliminating RLR and MAVS signaling via conditional MAVS knockout in CD11c^+^ cells, but not in B cells, results in very similar results as completely deleting MAVS including elevations of virus titers, Ab levels and T cell responses after WNV infection (Figs 4 and 5). Thus, signaling via MAVS in DCs and not in B cells is responsible for regulating high affinity Ab responses to WNV. Consistent with our data, it has been demonstrated that the selective deletion of IFNAR on CD11c^+^ cells results in uncontrolled WNV replication as well as heightened pro-inflammatory cytokine responses [32]. Clearly, the absence of MAVS or IFNAR in this critical DC population restricts the induction of antiviral immune responses allowing for uncontrolled virus replication [8,30,33]. On the other hand, enhanced virus replication can result in overactive inflammation. When IFNAR^fl/fl^CD11c^Cre+^ mice were infected with WNV, for example, serum pro-inflammatory cytokines including IL-6, TNF-α and IL-1β were all significantly elevated, which resulted in septic shock [32]. Similarly, MAVS^-/-^ mice experience elevated levels of inflammation following WNV-TX infection [9]. In particular, WNV induces a greater expansion of CD11c^+^ Ly6C^hi^ inflammatory monocytes/DCs in draining lymph nodes, spleens and the CNS, and increased levels of inflammatory cytokines in serum in the absence of MAVS signaling [9]. Also, RIG-I can associate with the adaptor protein ASC to form a MAVS-independent and NLR-independent RIG-I inflammasome complex that activate caspase-1 and induce IL-1β and IL-18 release [3]. Therefore, a possible consequence of the absence of the MAVS signaling pathway in DCs, could be that the other RIG-I-mediated RNA virus-induced pro-inflammatory pathways are enhanced. It is likely that some inflammatory cytokines induced in the absence of MAVS, such as IL-6, promote B cell differentiation and Ab responses. IL-6, for example, is also a critical cytokine in the activation and differentiation of T_FH_ cells [34]. Furthermore, it is possible that under elevated levels of both Ag and inflammation either more antigen presenting cells are activated and/or activated antigen presenting cells become more efficient at presenting Ag and stimulating T cells. Both of these scenarios would result in elevated adaptive immune responses.

Our study highlights that MAVS expressed by CD11c^+^ DCs restrains viral replication, B cell activation and humoral responses during virus infection. More studies are needed to determine the physiological relevance and the mechanisms underlying this phenomenon. It remains to be established whether enhanced humoral immunity in the absence of MAVS expression by DCs is just a consequence of increased virus titers and/or other mechanisms involving a direct communication between DCs and B cells. Nonetheless, an intriguing possibility is that the regulation of B cell responses by MAVS/type I IFN signaling in CD11c^+^ DCs could be part of a feedback loop to avoid over activation of innate and adaptive immune responses and control pathological inflammation during viral infection [10,32,35].

DCs constitutively express members of the RLR signaling pathways, and viral RNA triggers MAVS activation, Type I/III IFN and an anti-viral ISG program in DCs [2,3,32,33]. Other novel sensors of cytosolic RNA like DHX33, have also been shown to activate MAVS-dependent signaling in DCs [36]. STING, another viral sensor highly expressed in DCs, potentiates RIG-I-mediated antiviral signaling in response to flavivirus infection by directly interacting with RIG-I and MAVS [37]. Thus, although the classical RIG-I/MDA5/ISG pathway may be a major player in the DC-dependent control of WNV replication, other novel MAVS-mediated pathways activated during WNV infection could also play a role.

## Supporting information

S1 Fig Generation and verification of MAVS conditional knockout mice. (A) Generation of a conditional allele, via a sequence replacement strategy to knock-out the *MAVS* gene. The construct contains loxP sites that flank exon 3, a 5 kb 5’ arm of homology (containing exon 2), a 5 kb 3’ arm of homology (containing exons 4–6), a Diphtheria Toxin A (DTA) cassette, and a Neomycin (Neo) cassette flanked by frt sites for selective deletion. The Neo element allowed for positive selection in ES cells, while the DTA element permitted negative selection in ES cells. After homologous recombination of the conditional knock-out construct, the PGK-Neo was excised via Flp-e administration. The *MAVS* gene had normal expression until Cre-mediated deletion of exon 3. This recombination created a frameshift mutation, resulting in a premature stop, which rendered the *MAVS* gene inactive. (B) qRT-PCR analysis of *Mavs* exon 3 to verify deletion under Cre control. Splenocytes from MAVS^*fl/fl*^, MAVS^*fl/fl*^CD11c^Cre+^, and MAVS^*fl/fl*^Mb1^Cre+^, were subjected to CD11c^+^ magnetic bead positive selection, followed by B cell isolation by magnetic bead negative selection. Cells were lysed and RNA extracted for qRT-PCR analysis.
(TIF)

S2 Fig Flow cytometry gating strategies. After gating out debris, doublets and dead cells, CD3^-^ cells were evaluated for B220 and CD138 expression. Plasmablasts were defined as CD3^-^, B220^lo^, CD138^+^, CD19^+^ and were either IgM^+^ or IgM^-^. Plasma cells, defined as CD3^-^, B220^lo^, CD138^+^, CD19^-^, were either IgM^+^ or IgM^-^. GC B cells were defined as CD3^-^, B220^+^, CD38^-^, GL7^+^. A separate panel was used to define subsets of CD3^+^ T cells. CD4 T follicular helper cells (T_FH_) were defined as CD3^+^, CD4^+^, CD44^+^, CD62L^-^, CXCR5^+^, PD-1^+^. CD8 T effector cells (T_EFF_) were defined as CD3^+^, CD8^+^, CD44^+^, CD62L^-^.
(TIF)

S3 Fig Time course of humoral responses in mice deficient in MAVS following WNV infection. WT and MAVS^-/-^ mice were infected with 100PFU WNV-MAD. (A) Splenic B220^lo^, CD138^+^ plasma cells were evaluated by flow cytometry. (B) WNVE-specific IgM and IgG Ab secreting cells (ASCs) from the spleen were analyzed by ELISPOT. (C) Splenic B220^+^, CD38^-^, GL7^+^ GC B cells were determined using flow cytometry. Data are from one experiment per time point. *p<0.05, **p<0.01, ***p<0.001, ****p<0.0001 by 2-way ANOVA with Sidak’s multiple comparison test.
(TIF)

S1 Table Flow cytometry antibodies. Clone and source of antibodies used for flow cytometry.
(XLSX)
