# Supplementary figures and images for: Dendritic cell-associated MAVS is required to control West Nile virus replication and ensuing humoral immune responses
Source: PLoS One. 2019 Jun 26;14(6):e0218928. doi: 10.1371/journal.pone.0218928 (PMC6594639; doi:10.1371/journal.pone.0218928)

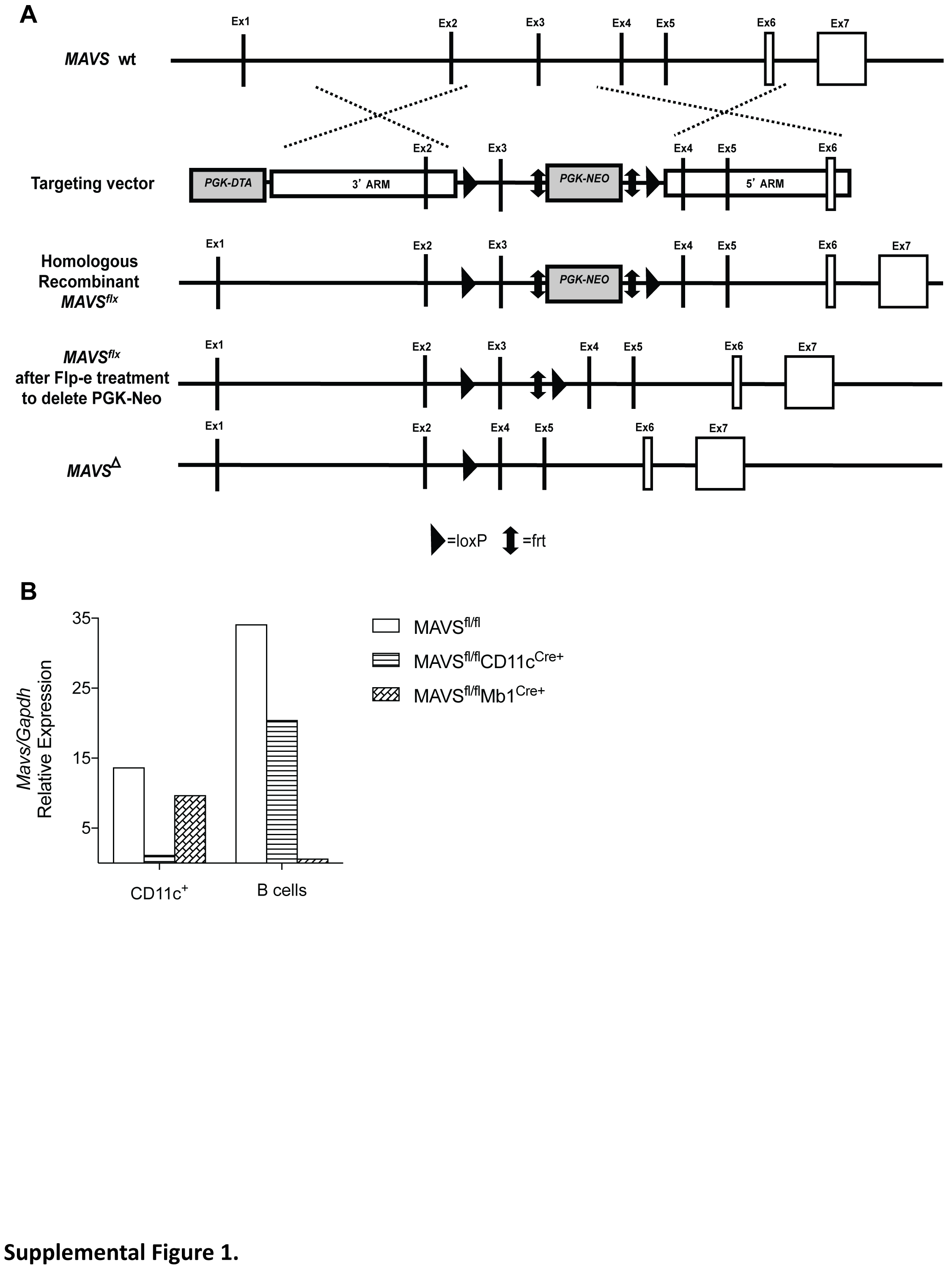

Supplement: S1 Fig — (A) Generation of a conditional allele, via a sequence replacement strategy to knock-out the MAVS gene. The construct contains loxP sites that flank exon 3, a 5 kb 5’ arm of homology (containing exon 2), a 5 kb 3’ arm of homology (containing exons 4–6), a Diphtheria Toxin A (DTA) cassette, and a Neomycin (Neo) cassette flanked by frt sites for selective deletion. The Neo element allowed for positive selection in ES cells, while the DTA element permitted negative selection in ES cells. After homologous recombination of the conditional knock-out construct, the PGK-Neo was excised via Flp-e administration. The MAVS gene had normal expression until Cre-mediated deletion of exon 3. This recombination created a frameshift mutation, resulting in a premature stop, which rendered the MAVS gene inactive. (B) qRT-PCR analysis of Mavs exon 3 to verify deletion under Cre control. Splenocytes from MAVSfl/fl, MAVSfl/flCD11cCre+, and MAVSfl/flMb1Cre+, were subjected to CD11c+ magnetic bead positive selection, followed by B cell isolation by magnetic bead negative selection. Cells were lysed and RNA extracted for qRT-PCR analysis. (TIF) [file pone.0218928.s001.tif]

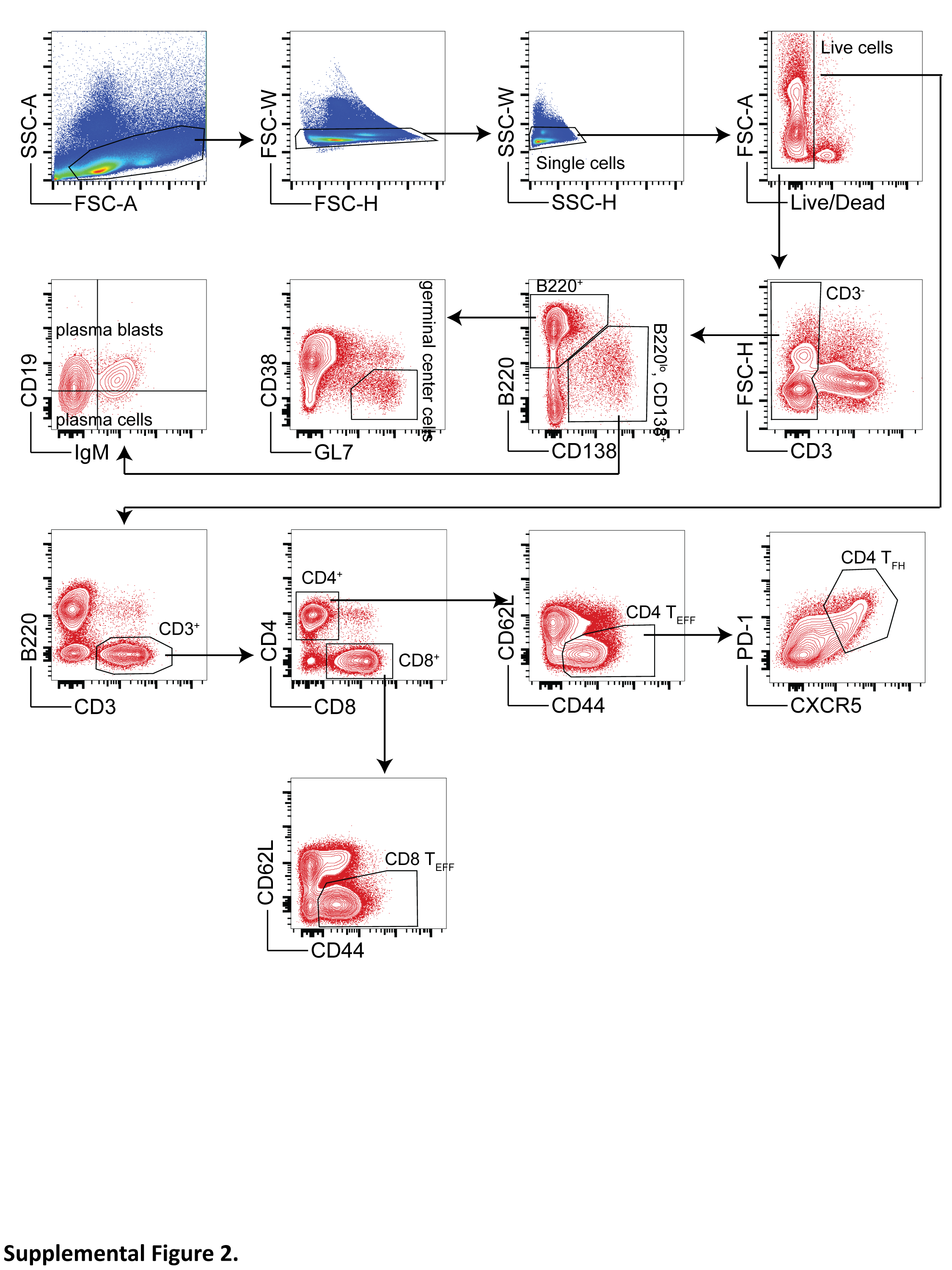

Supplement: S2 Fig — After gating out debris, doublets and dead cells, CD3- cells were evaluated for B220 and CD138 expression. Plasmablasts were defined as CD3-, B220lo, CD138+, CD19+ and were either IgM+ or IgM-. Plasma cells, defined as CD3-, B220lo, CD138+, CD19-, were either IgM+ or IgM-. GC B cells were defined as CD3-, B220+, CD38-, GL7+. A separate panel was used to define subsets of CD3+ T cells. CD4 T follicular helper cells (TFH) were defined as CD3+, CD4+, CD44+, CD62L-, CXCR5+, PD-1+. CD8 T effector cells (TEFF) were defined as CD3+, CD8+, CD44+, CD62L-. (TIF) [file pone.0218928.s002.tif]

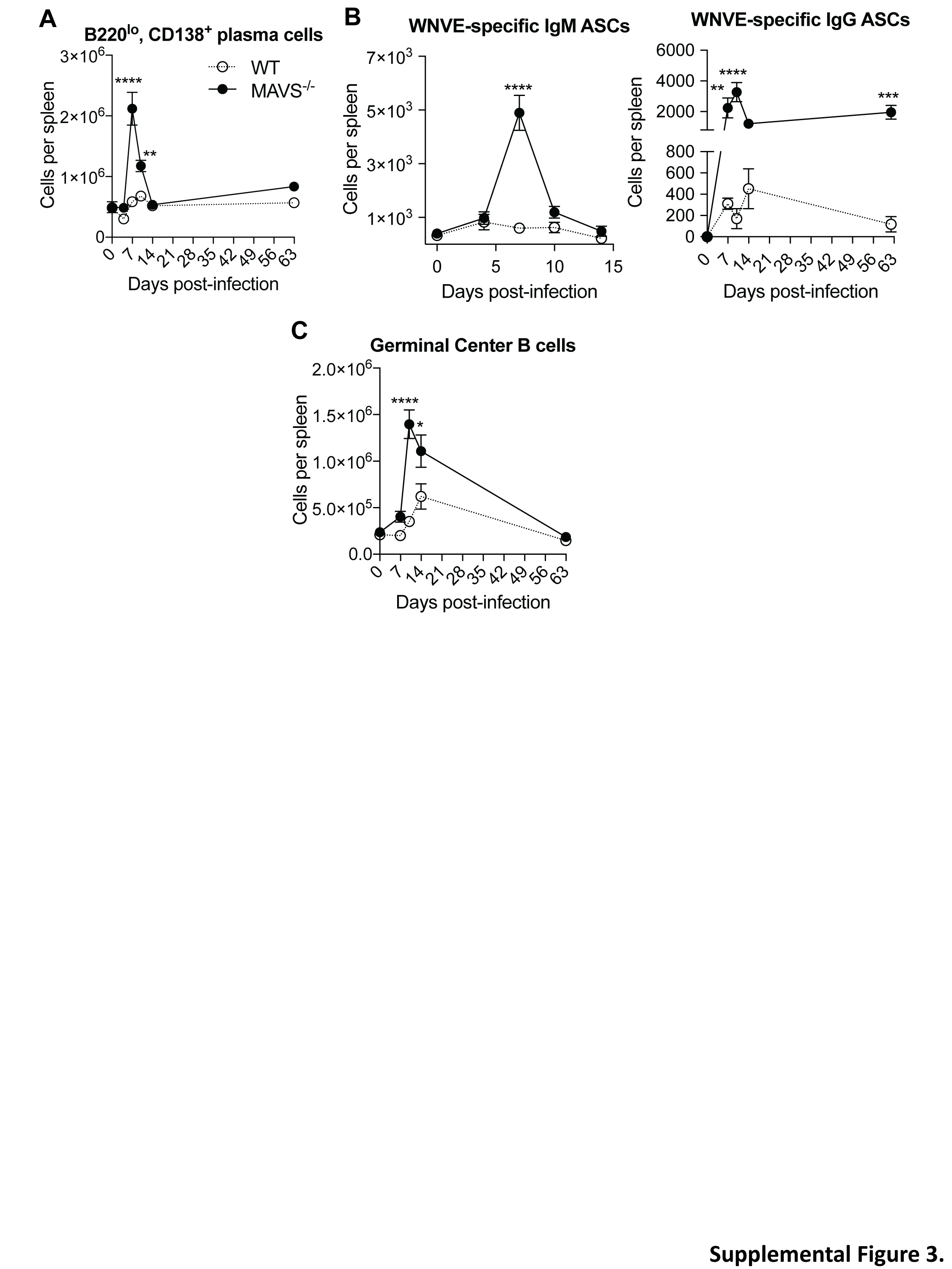

Supplement: S3 Fig — WT and MAVS-/- mice were infected with 100PFU WNV-MAD. (A) Splenic B220lo, CD138+ plasma cells were evaluated by flow cytometry. (B) WNVE-specific IgM and IgG Ab secreting cells (ASCs) from the spleen were analyzed by ELISPOT. (C) Splenic B220+, CD38-, GL7+ GC B cells were determined using flow cytometry. Data are from one experiment per time point. *p<0.05, **p<0.01, ***p<0.001, ****p<0.0001 by 2-way ANOVA with Sidak’s multiple comparison test. (TIF) [file pone.0218928.s003.tif]
